# Marked Rebound of Platelet Count in the Early Postpartum Period in a Patient with Essential Thrombocythemia

**DOI:** 10.1155/2021/6633790

**Published:** 2021-02-02

**Authors:** Yoshinori Hashimoto, Rina Hosoda, Hiromi Omura, Takayuki Tanaka

**Affiliations:** Department of Hematology, Tottori Prefectural Central Hospital, Tottori, Japan

## Abstract

Essential thrombocythemia (ET) occurs predominantly in the elderly, but approximately 20% of patients are <40 years old. Unlike other myeloproliferative neoplasms, ET occurs more commonly in women. We encountered a 38-year-old women diagnosed with ET who exhibited elevated platelet count in early pregnancy. Her platelet count exceeded 1500 × 10^9^/L by late pregnancy; interferon *α* was administered but failed to induce an adequate response. She underwent emergency cesarean delivery at 37 weeks of gestation. Although her platelet count was 1000 × 10^9^/L immediately after delivery, it markedly increased to 3271 × 10^9^/L approximately 2 weeks later. Cytoreductive therapy was resumed; the subsequent course was free from complications. Several review articles have indicated that because platelet counts of patients may again increase to the pregnancy level or rebound after delivery, cytoreductive therapy should be administered if necessary. However, there is insufficient information on when therapeutic interventions are necessary and how they should be performed. It remains unknown whether the platelet count will decrease after some time without treatment if it rebounds. We hope management guidelines will be established by collecting detailed data on the postpartum course as well as during pregnancy.

## 1. Introduction

Essential thrombocythemia (ET) occurs predominantly in the elderly, but approximately 20% of patients are <40 years old [1]. Unlike other myeloproliferative neoplasms (MPNs), ET occurs more commonly in women. Consequently, encountering ET in women of reproductive age is not a rare occurrence. Pregnancy and delivery may promote thromboembolism; thus, the perinatal management of patients with ET is becoming an important issue. Although expert consensus and several guidelines describing the management of these patients have been reported [2–6], there are currently no clear guidelines in Japan [7]. Some of the aforementioned guidelines recommend interferon (IFN) therapy for pregnant women with ET who are at a high risk of pregnancy loss. However, IFN is not approved for patients with ET in Japan. Furthermore, because ET is likely to cause thrombotic events, particularly in the postpartum period, low-molecular-weight heparin is commonly administered for 6 weeks after delivery in Europe and the United States. However, it is not indicated for such use in Japan, either. According to aforementioned and other guidelines, the platelet counts of patients with ET increase after delivery [3–6], but many aspects regarding the timing and degree of rebound remain unknown. Few guidelines, including Japanese guidelines [7], mention the optimal blood test interval and therapeutic strategies.

We present a case in which IFN therapy was administered when the platelet count exceeded 1500 × 10^9^/L during late pregnancy. In the present case, the platelet count decreased to approximately 1000 × 10^9^/L immediately after delivery, and cytoreductive therapy was discontinued. However, approximately 2 weeks later, the platelet count markedly rebounded.

## 2. Case Presentation

The patient was a 38-year-old woman who was referred to our hospital at approximately 7 weeks of gestation for an elevated platelet count detected during a prenatal checkup. She had vaginally delivered her first child at 40 weeks of gestation 8 years earlier and her second child at 39 weeks of gestation 5 years earlier. No abnormalities were observed during the perinatal periods of both pregnancies, and both the patient and her infants were healthy. She had no history of thrombosis or cardiovascular risk factors, such as diabetes mellitus, hypertension, or dyslipidemia, and was a nonsmoker. At her visit, the physical examination failed to identify any abnormal findings or splenomegaly. Blood tests indicated a white blood cell count of 11.4 × 10^9^/L, a hemoglobin level of 14.0 g/dL, a hematocrit level of 40.3%, a platelet count of 1074 × 10^9^/L, and a von Willebrand factor ristocetin cofactor activity (vWFRCo) of 49% (Table 1). At 9 weeks of gestation, a bone marrow biopsy was performed that revealed normocellular bone marrow with an increased number of large to giant megakaryocytes and absence of evidence of reactive thrombocytosis; a diagnosis of ET was made. The presence of driver gene mutations was evaluated, and the patient was negative for all Janus kinase 2 (JAK2V617F), calreticulin (CALR), and myeloproliferative leukemia (MPL) mutations, which suggested that she had the so-called triple-negative ET.

**Figure 1:**
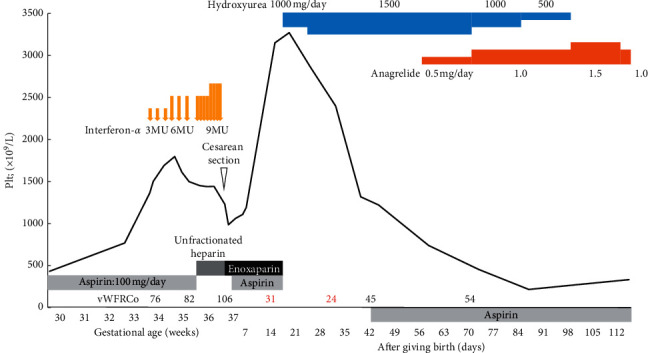
Clinical course and serial changes in the platelet count. MU, million units; VWFRCo, von Willebrand factor ristocetin cofactor activity.

Low-dose aspirin was initiated. Her platelet count decreased as the pregnancy progressed. At 30 weeks of gestation, her platelet count had decreased to 432 × 10^9^/L. Subsequently, her platelet count rapidly rebounded. At 34 weeks of gestation, it exceeded 1500 × 10^9^/L, which suggested that she was at a high risk (Figure 1). We provided sufficient explanation to the patient and obtained informed consent from her. After the approval of the ethics committee of our hospital, IFN*α* (Sumiferon^®^ Dainippon Sumitomo, Osaka, Japan) was administered at 34 weeks and 2 days of gestation. IFN*α* was administered at a dose of 3 million units 3 times/week at 34 weeks of gestation and at a dose of 6 million units 3 times/week at 35 weeks. The decrease in the platelet count was insufficient; thus, IFN*α* was administered at a dose of 6 million units daily beginning at 36 weeks, and the daily dose was subsequently increased to 9 million units. Although no adverse events associated with IFN*α* were observed, her platelet count decreased to only 1229 × 10^9^/L. Ultimately, the patient underwent emergency cesarean delivery at 37 weeks and 1 day of gestation (low-dose aspirin was switched to unfractionated heparin at 36 weeks of gestation).

The infant weighed 2581 g and had Apgar scores of 7 points at 1 min and 8 points at 5 min. Although the infant exhibited transient tachypnea and was temporarily admitted to the neonatal intensive care unit, there were no apparent complications, and the infant was discharged from our hospital. The platelet count of the infant at birth was 317 × 10^9^/L. After delivery, low-molecular-weight heparin was initiated for the mother, and low-dose aspirin was also resumed. Her platelet count was approximately 1000 × 10^9^/L. Cytoreductive therapy was discontinued temporarily, and we allowed her to breastfeed as per her request. At 18 days after delivery, her platelet count had markedly increased to 3271 × 10^9^/L, and her vWFRCo decreased to 31%. She appeared to have acquired von Willebrand syndrome; thus, we determined that she was at a high risk of bleeding. Administration of low-molecular-weight heparin and low-dose aspirin and breastfeeding were discontinued, and hydroxyurea was initiated as a cytoreductive therapy. Approximately 2 months after delivery when her platelet count reached controllable levels, anagrelide was added. Her platelet count was well controlled; thus, hydroxyurea was discontinued after approximately 3 months of use. At present, her platelet count is controlled at approximately 400 × 10^9^/L with 1.0 mg/day of anagrelide. The results of the gene mutation test after cytoreductive therapy were negative for TET oncogene family number 2 (TET2), additional sex combs like 1 (ASXL1), isocitrate dehydrogenase ½ (IDH1/2), and tumor protein p53 (TP53) mutations.

## 3. Discussion

Both pregnancy and ET contribute to the risk of thrombosis; thus, pregnant women with ET may be at a higher risk of thrombosis. Additionally, various complications, such as placental infarction, fetal growth restriction, and fetal wastage, can affect not only the mother but also the fetus. Greiesshammer et al. identified case reports of ≥9 pregnancies in ≥4 patients with ET that were published in and after 2000 and analyzed 10 case reports describing pregnancy outcomes [4]. According to their analysis, the live birth rate in pregnant women with ET was almost 70%; the full-term normal delivery rate was lower in pregnant women with ET than in healthy pregnant women, and the rates of spontaneous abortion and stillbirth were higher. These findings cannot be disregarded. High-risk pregnancies in patients with MPN are often defined based on the definitions developed by Greiesshammer et al. [4] and the European LeukemiaNet [2]. Additionally, expert consensus and several guidelines recommend the use of IFN*α* for high-risk pregnancy [2–6], and the Japanese guidelines also indicate that the use of IFN*α* should be considered although it is not covered by the National Health Insurance [7]. A previous retrospective study reported that the use of IFN*α* is an independent factor affecting the live birth rate [8], and a recent systematic review and meta-analysis of pregnant women with MPN also showed that the use of IFN is associated with a high live birth rate [9].

Based on the above discussion, IFN*α* was used in our patient with due ethical considerations; unfortunately, its effect was insufficient. Her platelet count rapidly increased around 34 weeks of gestation despite increasing the IFN*α* dose; hence, the IFN*α* administration period might have been insufficient. Edahiro et al. reported that the median platelet count decreased by approximately 37% from 910 × 10^9^ to 573 × 10^9^/L in seven patients after 2 months of IFN*α* therapy that was started after the discovery of pregnancy [10]. As our patient received the drug for approximately 1 month, its effect might have been insufficient. Generally, IFN*α* is thought to exert favorable molecular genetic effects because it can target cells positive for not only JAK2V617F mutations but also CALR and other mutations [11]. However, a report of the use of pegylated IFN*α* in patients positive for CALR mutations showed that its molecular genetic effects were lower in patients with CALR mutations and in those with mutations of additional genes such as TET2, ASXL1, IDH2, and TP53 [12]. In other words, the sensitivity of IFN*α* may differ among mutated clones. Moreover, a small-scale study suggests that patients with triple-negative ET may be more resistant to IFN*α* than patients with JAK2V617F or CALR mutations [10]. According to our tests, our patient did not have any abnormalities of the aforementioned additional genes, but she did have triple-negative ET. Consequently, the effects of IFN*α* might have been limited.

Finally, we would like to discuss the postpartum rebound of platelet count in our patient. Several review articles have indicated that because platelet counts of patients may again increase to the prepregnancy level [13] or rebound after delivery, cytoreductive therapy should be administered if necessary [3–6]. However, there is insufficient information on when therapeutic interventions are necessary and how they should be performed. It remains unknown whether the platelet count will decrease after some time without treatment if it rebounds. According to our searches in the English literature, some reports indicate that no particular cytoreductive therapy was required after delivery, whereas other reports describe cases in which patients were treated for a rebound of platelet count [14–18]. Sakai et al. reported a case in which the platelet count increased to ≥1000 × 10^9^/L within 3 months after delivery and another case in which the platelet count increased to ≥800 × 10^9^/L within 2 months after delivery. Cytoreductive therapy was resumed in the former case [14]. Iwashita et al. reported a case of a woman, para 2, whose platelet count increased to ≥2000 × 10^9^/L within 1-2 months after delivery in both pregnancies but decreased to approximately 800 × 10^9^ to 900 × 10^9^/L several months later [15]. Although the prepregnancy platelet count affects the postpartum count, one report cited a platelet count increase to a maximum of 3000 × 10^9^/L after delivery [19]. However, we did not identify any patients in the literature with a postpartum platelet count increasing as high as that of our patient. After pregnancy, cytoreductive therapy is rarely required because young women are often at a low risk based on the conventional risk classification for thrombosis [20]. Furthermore, the decision to perform cytoreductive therapy is expected to differ depending on whether the patient will breastfeed their infant after delivery. Thus, individualized treatment should be considered. While we assume that treatment varies depending on the postpartum status of the patients, a certain expert consensus indicates that blood tests should be counted at 6 weeks after delivery [3]. However, caution should be exercised because some women exhibit a rebound of platelet count within 2–4 weeks after delivery, as with our patient and the aforementioned cases [16, 17].

In conclusion, we encountered a patient who exhibited a marked rebound of platelet count soon after delivery. She resumed cytoreductive therapy; the subsequent course was uneventful without any complications. The evidence for the treatment of pregnant women with ET is limited. We hope that management guidelines will be established through the collection of data both during pregnancy and detailed data on the postpartum course.

## Figures and Tables

**Table 1:** Laboratory findings on admission.

*Peripheral blood*	
WBC	11.4 × 10^9^/L
Neu	76.5%
Lym	17.1%
Mon	4.9%
Eos	1.4%
Bas	1.0%
RBC	4.6 × 10^12^/L
Hb	14.0 g/dL
Hct	40.3%
MCV	86.9 fL
Ret	1.8 × 10^12^/L
Plt	1074 × 10^9^/L

*Chemistry*	
TP	7.5 g/dL
Alb	4.8 g/dL
T-bil	0.6 mg/dL
AST	18 U/lL
ALT	25 U/L
ALP	117 U/L
LDH	163 U/L
BUN	7.2 mg/dL
Cr	0.4 mg/dL
UA	3.2 mg/dL
Na	135 mEq/L
K	4.3 mEq/L

*Coagulation*	
PT-INR	0.95
APTT	30.2 sec
vWFRCo	49%

*Serology*	
CRP	0.04 mg/dL

*Other findings*	
JAK2V617F	<1.0%
MPLW515L	(−)
MPLW515K	(−)
CALR type1	<1.0%
CALR type2	<1.0%
ABO/Rh	A/+

Ret, reticulocyte; VWFRCo, von Willebrand factor ristocetin cofactor activity; JAK2, Janus kinase 2; MPL, myeloproliferative leukemia protein; CALR, calreticulin, ABO/Rh, ABO blood group/Rhesus blood group.

## Data Availability

No data were used in this study.
